# β‐arrestin 2 negatively regulates NOD2 signalling pathway through association with TRAF6 in microglia after cerebral ischaemia/reperfusion injury

**DOI:** 10.1111/jcmm.14223

**Published:** 2019-02-22

**Authors:** Lin Chen, Lingjun Kong, Xinbing Wei, Yimeng Wang, Bing Wang, Xiumei Zhang, Jinpeng Sun, Huiqing Liu

**Affiliations:** ^1^ Department of Pharmacology School of Basic Medical Sciences Shandong University Jinan Shandong P.R. China; ^2^ Department of Emergency The people's Hospital of Huaiyin Jinan Shandong P.R. China; ^3^ Key Laboratory Experimental Teratology of the Ministry of Education Department of Biochemistry and Molecular Biology School of Medicine Shandong University Jinan P.R. China

**Keywords:** β‐arrestin 2, cerebral ischaemia/reperfusion, inflammation, NOD2

## Abstract

We previously reported that nucleotide‐binding oligomerization domain‐containing protein (NOD) 2 was involved in the inflammatory responses to cerebral ischaemia/reperfusion (I/R) insult. However, the mechanism by which NOD2 participates in brain ischaemic injury and the regulation of NOD2 in the process are still obscure. Increased β‐arrestin 2 (ARRB2) expression was observed in microglia following cerebral I/R in wild‐type mice besides the up‐regulation of NOD2 and TRAF6. Stimulation of NOD2 by muramyl dipeptide (MDP) in BV2 cells induced the activation of NF‐κB by the phosphorylation of p65 subunit and the degradation of IκBα. Meanwhile, the protein level of Cyclooxygenase‐2 (COX‐2), the protein expression and activity of MMP‐9 were significantly increased in BV2 cells after administration of MDP. Furthermore, overexpression of ARRB2 significantly suppressed the inflammation induced by MDP, silence of ARRB2 significantly enhanced the inflammation induced by MDP in BV2 cells. In addition, we observed endogenous interaction of TRAF6 and ARRB2 after stimulation of MDP or cerebral I/R insult, indicating ARRB2 negatively regulates NOD2‐triggered inflammatory signalling pathway by associating with TRAF6 in microglia after cerebral I/R injury. Finally, the in vivo study clearly confirmed that ARRB2 negatively regulated NOD2‐induced inflammatory response, as ARRB2 deficiency exacerbated stroke outcomes and aggravated the NF‐κB signalling pathway induced by NOD2 stimulation after cerebral I/R injury. These findings revealed ARRB2 negatively regulated NOD2 signalling pathway through the association with TRAF6 in cerebral I/R injury.

**Abbreviations**
NOD2: nucleotide‐binding oligomerization domain 2
ARRB2: β‐arrestin 2
ARRB2‐/‐: β‐arrestin 2 deficiet
I/R: ischaemia/reperfusion
MCAO: middle cerebral artery occlusion
MDP: muramyl dipeptide
NF‐κB: nuclear factor κB
WT: wild type
TRAF6: TNFR‐associated factor 6
TLRs: Toll‐like receptors

## 1 INTRODUCTION

Ischaemic stroke is an important cause of death and disability in adults worldwide with a complex sequence of pathophysiological events.^1^ An acute and sustained inflammatory response induced by cerebral ischaemia and reperfusion (I/R) is an important feature of the injury cascade.^2^ Innate immunity is the first line of defence against infection which is also associated with a number of sterile inflammatory events, such as cerebral ischaemic injury. Upon stroke, dying cellular debris which is recognized as damage‐associated molecular patterns (DAMP) activate Toll‐like receptors (TLRs) and nucleotide‐binding oligomerization domain (NOD)‐like receptors (NLRs) to induce inflammatory responses.^3^ NOD2, an important member of the NLR family, has been confirmed participating in inflammatory diseases by activating caspase recruitment domain (CARD), signal to the nuclear factor κB (NF‐κB).^4^ Moreover, we have been proved NOD2 contributed to cerebral injury by NOD2‐mediated inflammatory signalling in mice after ischaemic stroke.^5^ However, there is still a limited understanding about the regulatory mechanism of NOD2 on the inflammation of cerebral I/R.

β‐arrestins were adaptor proteins which terminate G protein activation by desensitizing and internalizing G‐protein‐coupled receptors (GPCRs).^6^ More recently, β‐arrestins have been identified as scaffold proteins binding with various target molecules and thus regulating a wide range of biological processes.^7^, ^8^ Growing evidence shows that β‐arrestin 2 (ARRB2) play a critical regulatory role in the inflammatory response.^9^ Li et al reported ARRB2 acts as an anti‐inflammatory endogenous agent in experimental arthritis^10^ and recent data suggested that ARRB2 negatively regulates polymicrobial sepsis‐induced inflammation.^11^ Moreover, ARRB2 plays a prominent role in the negative regulation of TLR4‐triggered inflammatory responses by regulating p38 MAPK function^12^ and ARRB2 interacted with NLRP3 to inhibit inflammation after traumatic brain injury.^13^ However, specific molecular mechanism involved in the regulation of ARRB2 in NOD2‐induced inflammation in cerebral I/R injury remains poorly understood. Here, we demonstrated that ARRB2 negatively regulated NOD2 signalling pathway through the association with TRAF6 in microglia after cerebral I/R injury.

## 2 MATERIALS AND METHODS

### 2.1 Drugs and reagents

2,3,5‐triphenyltetrazolium chloride (TTC) was purchased from Sinopharm Chemical Reagent Co (Shanghai, China). All chemicals and reagents used in this study were of analytical grade and were purchased from Sigma (St Louis, MS, USA) unless otherwise mentioned.

### 2.2 Mice

β‐arrestin 2 knockout (ARRB2^−/−^) mice on a C57BL/6 background were kindly provided by Dr. Jinpeng Sun, Shandong University. The wild‐type (WT) C57BL/6 mice were purchased from Laboratory Animals Center of Shandong University. All aspects of the animal care and experimental protocols were approved by the Committee on the Ethics of Animal Experiments of the Shandong University.

### 2.3 Animal models for transient focal cerebral ischaemia

Transient middle cerebral artery occlusion (MCAO) was induced in both ARRB2^−/−^ and WT mice (25‐28 g) as described previously.^5^ All surgeries were performed under sodium pentobarbital anaesthesia and all efforts were made to minimize suffering. After 2 hours of MCAO, reperfusion was initiated by the thread careful withdrawal. As an extrinsic ligand of NOD2, muramyl dipeptide (MDP, Sigma‐Aldrich, A9519) was intraventricularly administered to mice 30 minutes before MCAO. MDP (200 μmol/L in normal saline [NS]) or the vehicle (NS) was injected using a Hamilton syringe (3 μL, 0.4 mm posterior and 1.0 mm lateral to the bregma and 2.2 mm from the duramater). After reperfusion, the mice were anaesthetized, perfused with NS and then decapitated. The brains were immediately frozen in liquid nitrogen for the following studies.

### 2.4 Cell culture and treatment

Microglia cell line BV2 cells were cultured in the Dulbecco's modified Eagle's medium (DMEM, Gibco Carlsbad, CA, USA) supplemented with 10% foetal bovine serum (FBS, Gibco Carlsbad, CA, USA) at 37°C with 5% CO_2_. BV2 cells were stimulated by 2 μg/mL MDP for indicated time.

### 2.5 Cell transfection

Non‐specific scrambled shRNA and ARRB2 targeting shRNA, ARRB2 full‐length GFP vector and the negative control vector were obtained from Dr. Deling Yin, Department of Internal Medicine, College of Medicine, East Tennessee State University. Transfection was performed using lipofectamine 2000 reagent (Invitrogen Corporation, Carlsbad, CA, USA).

### 2.6 Western blot

Western blot was performed as described previously.^14^ Briefly, total proteins were isolated from mouse penumbral cortex and BV2 cells with RIPA buffer (Beyotime, Haimen, China). Protein concentrations were determined using a BCA Protein Assay reagent kit (Beyotime, Haimen, China). The primary antibodies used in this study were as follows: ARRB2 (Santa Cruz Biotechnology Cat# sc‐13140, Dallas, TX, USA), TRAF6 (Santa Cruz Biotechnology Cat# sc‐33897), NOD2 (Proteintech Group Cat# 66710‐1‐Ig, Wuhan, China), β‐Actin (Bioworld Cat# AP0060, Nanjing, China), GAPDH (Santa Cruz Biotechnology Cat# sc‐59541), NF‐κB p‐P65 (Cell Signaling Technology Cat# 3039, Danvers, MA, USA), IκBα (Cell Signaling Technology Cat# 9242L), COX‐2 (Proteintech Group Cat# 12375‐1‐AP), MMP‐2 (Proteintech Cat# 10373‐2‐AP) and MMP‐9 (Proteintech Group Cat# 10375‐2‐AP). The signals were quantified by scanning densitometry and data within a linear range were quantified using Image Quant software (GE Amersham, Piscataway, NJ, USA).

### 2.7 Immunofluorescence staining

Staining was performed on brain sections as described in our previous publication.^5^ In brief, sections were blocked by 10% normal donkey serum, and incubated in specific primary antibodies as follows: mouse anti‐ARRB2 (1:100 dilution), rabbit anti‐Iba1 (1:150 dilution) and rabbit anti‐TRAF6 (1:100 dilution). After being washed three times by PBS, the cells were incubated with corresponding Alexa 488‐conjugated donkey antimouse (1:200 dilution) and Alexa 555‐conjugated donkey anti‐rabbit (1:200 dilution) (Invitrogen, Gaithersburg, MD, USA) secondary antibodies. DAPI (Beyotime, Haimen, China) was used to stain nuclei. Images were obtained by confocal microscopy (Zeiss, Oberkochen, Germany). Quantification of the colocalization coefficient between ARRB 2 and TRAF6 displayed as Pearson coefficients in the colocalized volume (1, perfect correlation; 0, no correlation; −1, perfect inverse correlation).^15^

### 2.8 Co‐Immunoprecipitation

Total proteins were isolated from mouse penumbral cortex and BV2 cells with NP‐40 lysis buffer (Beyotime, Haimen, China). After centrifugation at 13 000 rpm at 4°C for 10 minutes, supernatants were collected and incubated under rotation with respectively 10 μL anti‐TRAF6 antibody in all samples at 4°C overnight. Ten microlitres of antimouse‐IgG (Santa Cruz Biotechnology, Inc., USA) was added to another simple stimulated by 30 minutes to serve as a negative control. Next day, 20 μL Protein A + G agarose (Beyotime, Haimen, China) was put into the mixture and incubated for 2 hours at 4°C. After incubation, bead‐linked immune complexes were washed five times with NP‐40 buffer (centrifuged at 4°C at 3000 rpm for 5 minutes), eluted by boiling in 2 × loading buffer (Beyotime, Haimen, China) and analysed by western blot.

### 2.9 Gelatin zymography

Gelatin zymography was performed to investigate the activity of matrix metalloproteinases (MMPs). BV2 cell supernatants for detecting were collected and centrifuged at 12 000 rpm at 4°C for 10 minutes to remove cell debris. Zymography was implemented with MMPs zymography electrophoresis kits (Genmed, Shanghai, China) according to the manufacture's protocols. Experiments were first performed with nondenaturing electrophoresis through an 8% SDS‐PAGE polyacrylamide gels with 0.1% gelatin (Sigma‐Aldrich, St. Louis, MO, USA). After electrophoresis, the 10 ×  Zymogram Renaturing Buffer was diluted to 1:9 and gels were incubated in the buffer (10 ml for each gel) with gentle agitation for 1 hour at room temperature. One hour later, the incubation solution was replaced with 1 ×  Zymogram Developing Buffer (10 mL for each gel) and then incubated at 37°C for at least 40 hours with gentle agitation. After 37°C incubation, gels were stained with Coomassie Blue solution for 1 hour and destained with destaining buffer for 1 hour. Areas of MMPs will show up as clear bands and detected the signals by *Bio‐Rad* ChemiDoc XRS + (BIO‐RAD, Inc., Hercules, CA, USA).

### 2.10 Neurological function and infract volume assessment

Twenty‐four hours after I/R a 4‐tiered neurological scoring system and infarct volume were used to determine the outcome by a blinded observer as described previously.^16^ Postural reflex was scored on a four‐point grade scale: 0, normal function; 1, flexion of the torso and contralateral forelimb on lifting the animal by the tail; 2, circling to the contralateral side but normal posture at rest; 3, reclination to the contralateral side at rest; and 4, absence of spontaneous motor activity. TTC infarct measurement techniques were performed to measure infarct size. The brains were sliced into 2 mm thick coronal sections for staining with 2% TTC in phosphate buffer saline at 37°C for 20 minutes. Infarction volume was measured by digital imaging (Digital Camera, Olympus MDF‐382E) and image analysis software (Image‐Pro Plus Version 6.0). The infarct area was calculated across each section and was presented as a percentage relative to the area of the contralateral hemisphere.

### 2.11 Statistical analysis

All data were reported as mean ± SEM and analysed with GraphPad 6.0 Software. One‐way analysis of variance (ANOVA) followed by Tukey's multiple comparisons test was used to evaluate where differences among groups existed. *P* < 0.05 was considered statistically significance.

## 3 RESULTS

### 3.1 The ARRB2 expression was increased in WT mice subjected to cerebral I/R injury

**Figure 1:**
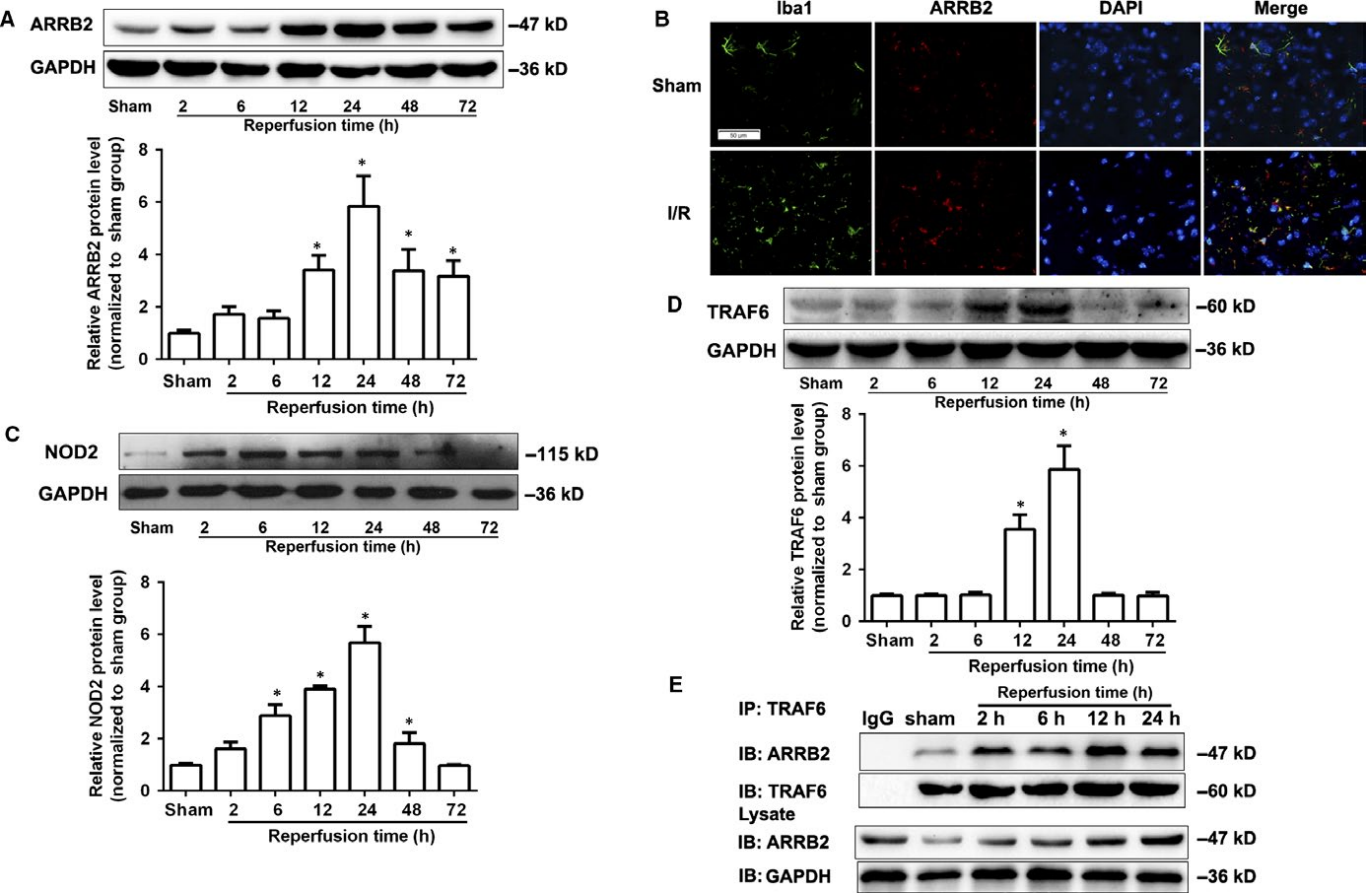
The expression of β‐arrestin2 (ARRB2) in wild‐type (WT) mice was increased after cerebral ischaemia‐reperfusion (I/R) injury. Western blot analysis of ARRB2 (A), NOD2 (C) and TRAF6 (D) protein levels in the penumbral cortex from WT mice after 2 h occlusion of the middle cerebral artery (MCAO) and 2, 6, 12, 24, 48 and 72 h reperfusion. Results are representative of six independent experiments. *P < 0.05 compared with sham group. (B) Representative images of double immunolabelling for ARRB2 and Iba‐1(microglia) in the penumbral cortex from WT mice after 2 h MCAO and 24 h reperfusion. DAPI indicates 4′,6‐diamidino‐2‐phenylindole. Scale bars: 50 μm. (E) The interaction of ARRB2 and TRAF6 was determined by Co‐Immunoprecipitation (Co‐IP) in the penumbral cortex from WT mice after 2 h MCAO and 2, 6, 12 and 24 h reperfusion

In order to assess the change of ARRB2 in cerebral I/R injury, western blot and immunofluorescence analysis were used to determine the protein level of ARRB2. As shown in Figure 1A, ARRB2 protein level in the ischaemic penumbra was upregulated in WT mice subjected to 2 hours MCAO followed by 12, 24 and 48 hours reperfusion respectively and reached a peak expression at 24 hours. We further found that ARRB2 was significantly increased in microglia in the cortex at 24 hours after I/R by double immunofluorescence analysis (Figure 1B). Moreover, the expressions of NOD2 and TRAF6 increased dramatically in WT mice subjected to 2 hours MCAO followed by 12 and 24 hours reperfusion respectively (Figure 1C and 1D). Furthermore, we investigated the interaction of ARRB2 and TRAF6 by Co‐IP. The results showed that the interaction of ARRB2 and TRAR6 were induced after cerebral I/R in WT mice, and reached its peak at 12 hours and kept to 24 hours after reperfusion (Figure 1E). These data suggested ARRB2 may be involved in cerebral I/R injury by regulating the inflammation through interaction with TRAF6.

### 3.2 MDP stimulation activated NF‐κB/COX‐2/MMP‐9 signalling pathway in the microglia

**Figure 2:**
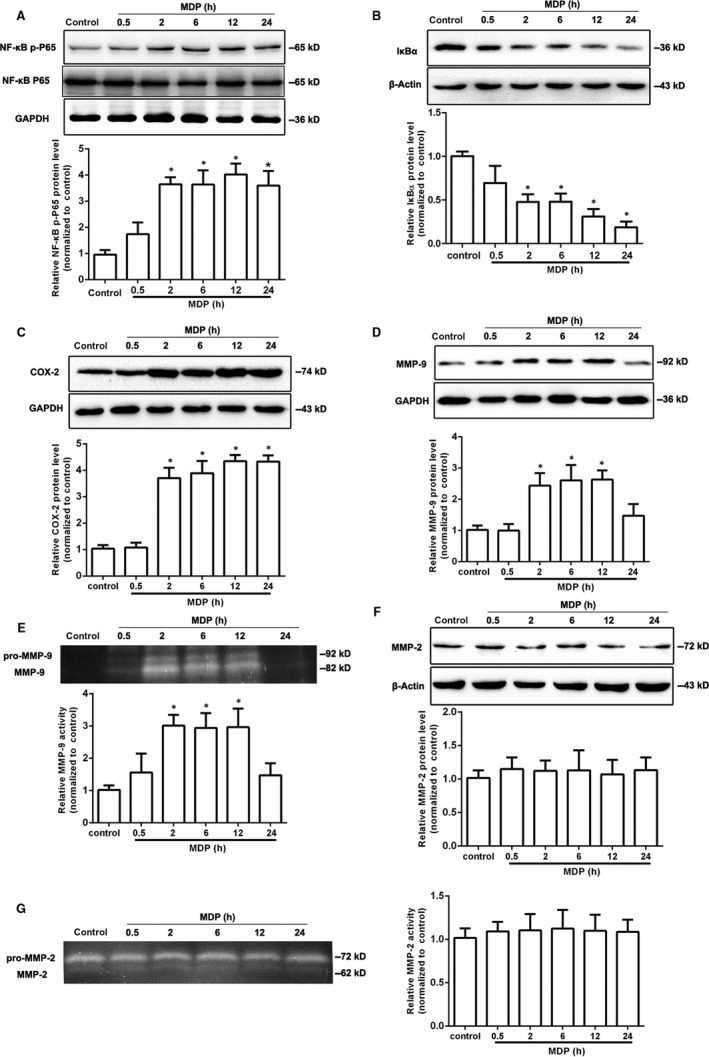
MDP stimulation activated NF‐κB/COX‐2/MMP‐9 signalling pathway in the microglia. BV2 cells were stimulated by 2 μg/mL MDP for 0.5, 2, 6, 12, 24 h. Western blot was used to detect the protein levels of NF‐κB p‐P65 *(*A), IκBα (B), COX‐2(C), MMP‐9 (D) and MMP‐2 (F). The activity of MMP‐9 *(*E) and MMP‐2 (G) was analysed by *Gelatin Zymography*. The values are mean ± SEM of three independent experiments. *P < 0.05 compared with the control group

Our previous study has been demonstrated that NOD2 participated in the inflammatory responses to cerebral I/R injury and significantly upregulated in microglia both in vivo and in vitro.^5^ To evaluate the exact function of NOD2 in microglia, we used MDP, an extrinsic ligand of NOD2 which triggers NOD2 and its downstream signalling pathway in macrophages. After BV2 cells were treated with 2 μg/mL MDP for 0.5, 2, 6, 12 and 24 hours, the NF‐κB pathway was markedly activated by the phosphorylation of p65 subunit and degradation in IκBα (Figure 2A,B). Meanwhile, the protein expression of COX‐2 and MMP‐9 were significantly increased at 2 hours and with a peak expression at 12 hours after MDP stimulation (Figure 2C,D). Furthermore, Gelatin Zymography results showed MDP stimulation dramatically activated MMP‐9 (Figure 2E). However, the protein level and activity of MMP‐2 did not changed obviously in microglia stimulated by MDP (Figure 2F,G). Collectively, these results suggested MDP stimulation activated NF‐κB/COX‐2/MMP‐9 signalling pathway in BV2 microglia cells.

### 3.3 ARRB2 negatively regulated NOD2 triggered inflammation in microglia

**Figure 3:**
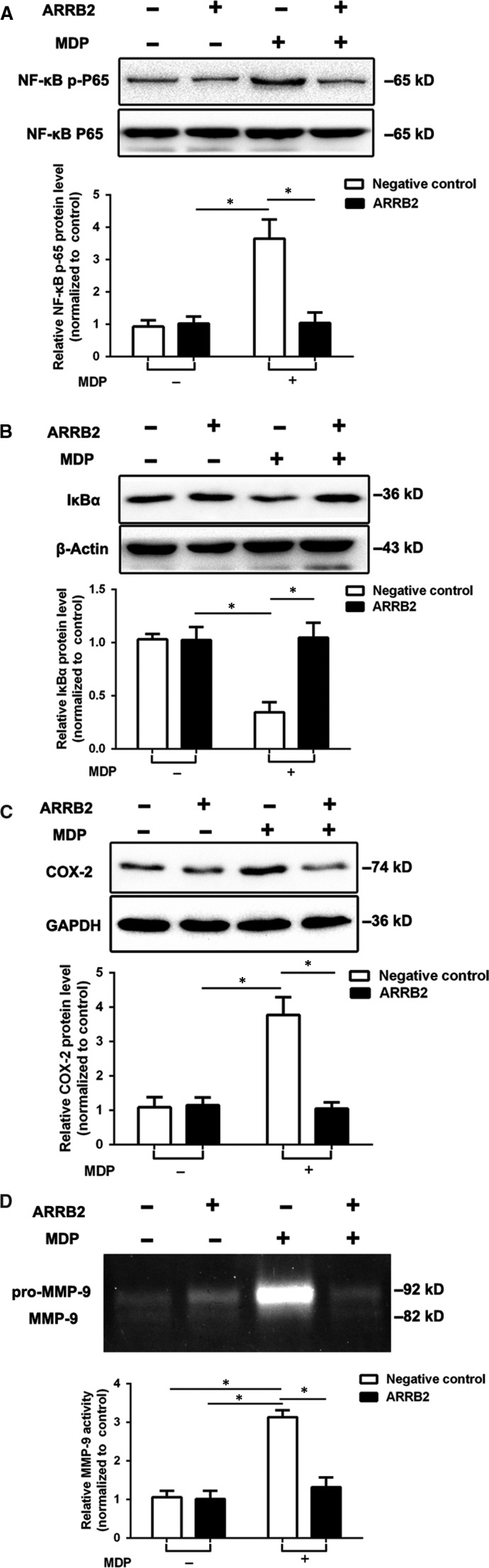
The overexpression of β‐arrestin2 (ARRB2) suppressed the NF‐κB/COX‐2/MMP‐9 signalling pathway activated by MDP stimulation. BV2 cells were transfected with either ARRB2 full‐length vector or control vector. Western blot were used to analyse for the protein levels of NF‐κB p‐P65 (A), IκBα (B) and COX‐2 (C) in BV2 cells after treated with 2 μg/mL MDP for 12 h. The activity of MMP‐9 (D) was analysed by *Gelatin Zymography*. Data are means ± SEM from three independent experiments. *P < 0.05 compared with indicated groups

**Figure 4:**
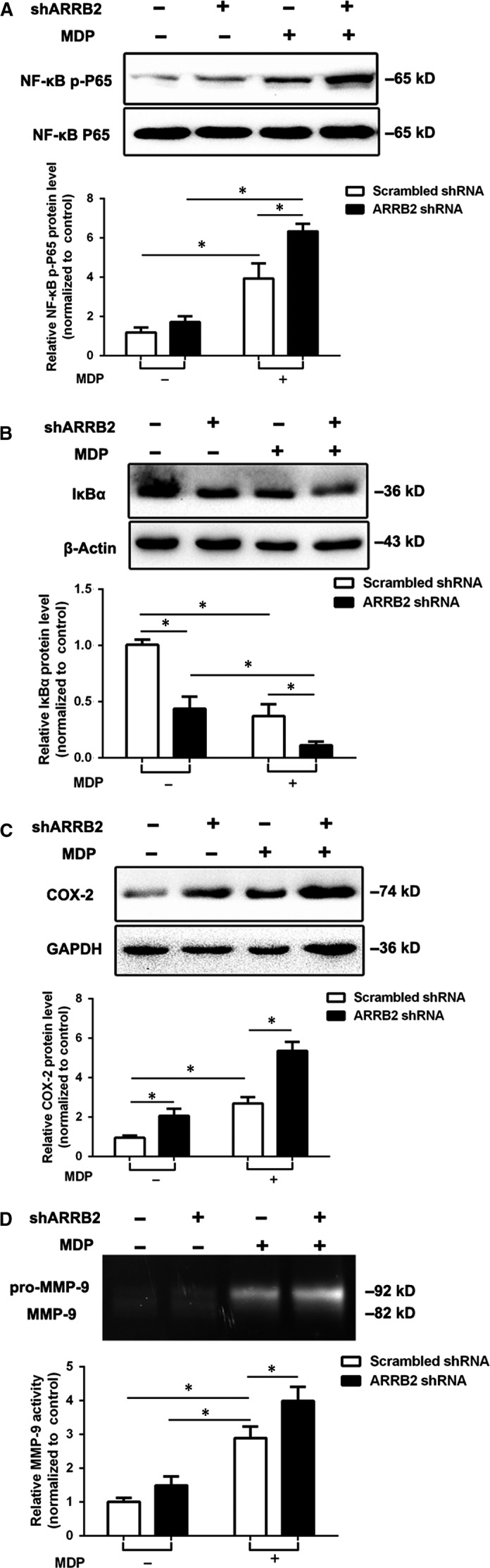
The deficiency of β‐arrestin2 (ARRB2) accelerated the NOD2 triggered NF‐κB/COX‐2/MMP‐9 signalling pathway. Scrambled shRNA or ARRB2 targeting shRNA were transfected into the BV2 cells. Western blot were used to analyse for the protein levels of NF‐κB p‐P65 (A), IκBα (B) and COX‐2 (C) in BV2 cells after treated with 2 μg/mL MDP for 12 h. The activity of MMP‐9 (D) was analysed by *Gelatin Zymography*. Data are means ± SEM from three independent experiments. *P < 0.05 compared with indicated groups

To assess the role of ARRB2 in NOD2‐induced inflammation, we transfected ARRB2 full‐length plasmid and shRNA ARRB2 plasmid to overexpress and silence this gene respectively in BV2 cells. Our results showed overexpression of ARRB2 significantly diminished the activation of NF‐κB induced by MDP in comparison with control vector group (Figure 3A,B), whereas silence of ARRB2 dramatically enhanced the activation of NF‐κB (Figure 4A,B). In addition, transfection of full‐length ARRB2 dramatically decreased the expression of COX‐2 (Figure 3C) and suppressed the activation of MMP‐9 (Figure 3D) induced by MDP. Meanwhile, ARRB2 deficiency significantly boosted the protein level of COX‐2 (Figure 4C) and the activation of MMP‐9 (Figure 4D). Taken together, our results imply ARRB2 negatively regulated NOD2 triggered NF‐κB/COX‐2/MMP‐9 signalling pathway in microglia cells.

### 3.4 MDP stimulation induced interaction of ARRB2 and TRAF6

**Figure 5:**
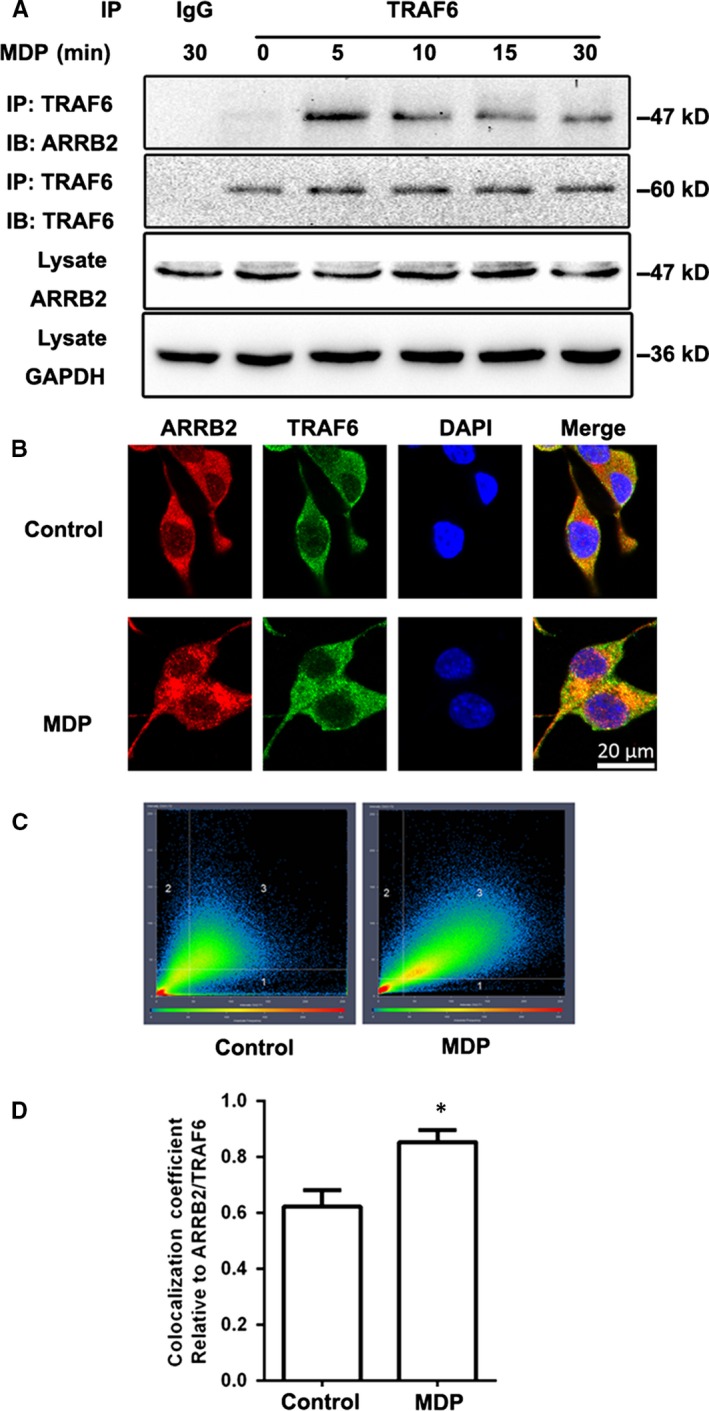
MDP stimulated interaction of β‐arrestin2 (ARRB2) with TRAF6. (A) BV2 cells were stimulated with MDP (2 μg/mL) for 5, 10, 15 and 30 min. Cell extracts were immunoprecipitated with anti‐TRAF6 antibody and then analysed together with cell lysate by immunoblotting with indicated primary antibodies. (B) BV2 cells were stimulated with MDP (2 μg/mL) for 5 min. ARRB2 (red) and TRAF6 (green) were viewed using confocal microscopy. Scale bars: 20 μm. (C) 2D fluorograms showing colocalization of ARRB2 and TRAF6 as a distribution of pairs of pixel intensities (with greater diagonal alignment correlating to higher colocalization). (D) Quantification of the colocalization coefficient between ARRB2 and TRAF6. *P < 0.05 compared with control group

Next, we explored how ARRB2 regulates NOD2‐induced inflammation. We used Co‐IP and confocal microscopy to monitor the interaction of ARRB2 and TRAF6, an upstream protein of NF‐κB in NOD2 signalling pathway. We observed that endogenous interaction of TRAF6 and ARRB2 reached a peak at 5 minutes after MDP stimulation (Figure 5A). Confocal immunofluorescence microscopy further confirmed the colocalization of ARRB2 and TRAF6 in MDP‐treated BV2 cells (Figure 5B–D). These data implicated that NOD2 activation leads to association of ARRB2 and TRAF6 in microglia cells, which negatively regulated the inflammation induced by MDP.

### 3.5 ARRB2 deficiency exacerbated stroke outcomes induced by NOD2 stimulation after cerebral I/R injury by aggravating inflammation in mice

**Figure 6:**
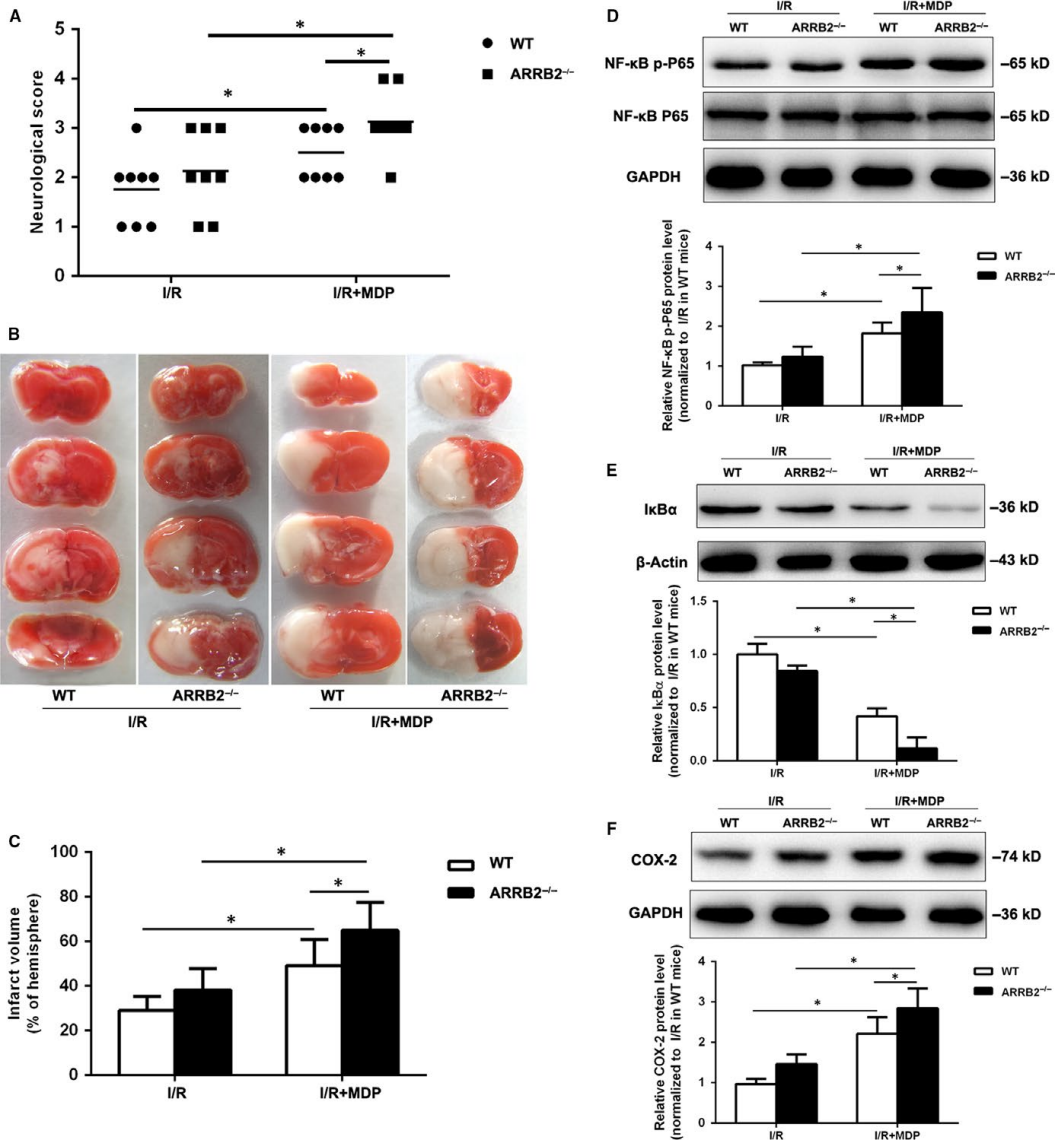
β‐arrestin2 (ARRB2) deficiency exacerbated stroke outcomes induced by NOD2 stimulation after cerebral ischaemia‐reperfusion injury. Wild type (WT) and β‐arrestin 2 deficient (ARRB2^−/−^) mice were subjected to 2 h MCAO and 24 h reperfusion. MDP, an extrinsic ligand of NOD2 was intraventricularly administered to mice 30 min before MCAO. (A) Neurological deficit scores (B) Representative photographs of coronal brain sections following infarction, stained with 2, 3, 5‐triphenyltetrazolium chloride (TTC). (C) Summary of cerebral infarct volume in brains. The infarct volume was expressed as the percentage of the contralateral hemispheric area. Western blot analysis of NF‐κB p‐P65 (D), IκBα (E) and COX‐2 (F) in the penumbral cortex. Results are representative of eight independent experiments. *P < 0.05 compared with indicated groups

We previously found that stimulation of NOD2 aggravated stroke outcomes.^5^ To evaluate the effect of ARRB2 on NOD2‐induced lesion in cerebral I/R, MDP the extrinsic ligand of NOD2 was intraventricularly administered 30 minutes before MCAO to WT and ARRB2^−/−^ mice. The results showed that administration of MDP in ARRB2^−/−^ mice resulted in a significant increase in neurological scores and infarct volume in comparison with WT mice. However, there were no statistical differences in the neurological scores and infarct volume between the WT and ARRB2^−/−^ mice without MDP administration (Figure 6A–C). Moreover, administration of MDP in ARRB2^−/−^ mice subjected to I/R exacerbated inflammatory response (Figure 6D–F). Taken together, our data indicated that ARRB2 deficiency aggravated stroke outcomes induced by NOD2 stimulation after cerebral I/R injury in mice by enhancing inflammation.

## 4 DISCUSSION

In this study, we identified that ARRB2 played a critical role in the negative regulation of NOD2‐induced inflammatory responses in cerebral I/R injury by interacting with TRAF6.

Inflammation is a key element in ischaemic stroke progression and innate immunity is considered to play pivotal role in the initiation of the inflammatory response in stroke and related injuries.^17^, ^18^ NOD2 is an important constituent in the innate immunity and inflammation. We recently proved that NOD2 was involved in the inflammatory responses to cerebral I/R insult and significantly upregulated in microglia both in vivo and in vitro.^5^ In this study, we further confirmed the inflammatory response mediated by NOD2 in microglia. Stimulation of NOD2 by MDP in BV2 cells induced the activation of NF‐κB by the phosphorylation of p65 subunit and the degradation of IκBα. NF‐κB activation is a classical signalling pathway of NOD2 which induces the transcription of many pro‐inflammatory genes.^19^ As a downstream of NF‐κB, COX‐2 is induced following brain ischaemia. Our previous study has been confirmed that NOD2 activation upregulated mRNA and protein expression of COX‐2 in plaques.^20^ We found that in BV2 cells the protein expression of COX‐2 was also significantly increased after administration of MDP. Scoditti et al reported that COX‐2 activity is associated with the regulation of matrix metalloproteinases (MMPs) a group of proteases involved in the breakdown of proteins of the extracellular matrix.^21^ Microglia is a major source of MMPs which are upregulated and activated following cerebral ischaemia.^22^ We found that both protein expression and activity of MMP‐9 were upregulated in BV2 cells after NOD2 stimulation whereas the protein expression and activity of MMP‐2 had no difference in this process. In fact, mice deficiency of MMP‐9, but not MMP‐2 had smaller infarcts compared to WT controls after focal cerebral ischaemia because MMP‐9 is produced by immune cells.^23^, ^24^, ^25^ Our results confirmed the speculation and indicated that NF‐κB/COX‐2/MMP‐9 signalling pathway is involved in NOD2‐mediated inflammation in BV2 cells. However, the mechanism involved in the regulation of NOD2‐mediated inflammation is still unclear.

Growing evidence indicates that ARRB2 modulates inflammation through multiple mechanisms^12^, ^26^. In present study, we confirmed ARRB2 was upregulated and localized in microglia after cerebral I/R injury. Since ARRB2 may play a critical regulatory role in inflammation, we postulated that the NOD2‐mediated inflammatory signalling pathway could be regulated by ARRB2. Here, we found that overexpression of ARRB2 significantly diminished the activation of NF‐κB, dramatically decreased the expression of COX‐2 and suppressed the activation of MMP‐9 induced by MDP. Conversely, silence of ARRB2 significantly enhanced the activation of NF‐κB/COX‐2/MMP‐9 signalling pathway induced by MDP in BV2 cells. It has been reported ARRB2 are essential negative regulators of innate immune activation by interact with TRAF6, preventing autoubiquitylation of TRAF6 and thus blocking the activation of NF‐κB and AP‐1 via TLR–IL‐1R signalling.^27^, ^28^ Furthermore, TRAF6 is also an important factor in the NOD2‐mediated NF‐κB inflammatory pathway.^29^ In our present study, the increased interaction between ARRB2 and TRAF6 indicated ARRB2 negatively regulates NOD2‐triggered inflammatory signalling pathway by associating with TRAF6 in microglia after I/R.

Wang et al also reported ARRB2 was upregulated in the peri‐infarct penumbra tissue in mouse model of cerebral ischaemia produced by MCAO, but ARRB2 deficiency cannot aggravate neuronal injury induced by cerebral ischaemia.^30^ Our result was consistent with this report. Moreover, we further confirmed that ARRB2 negatively regulated NOD2‐induced inflammatory response, as ARRB2‐deficient mice exacerbated stroke outcomes and deletion of ARRB2 aggravated the NF‐κB signalling pathway induced by NOD2 stimulation after cerebral I/R injury. Collectively, our study revealed that ARRB2 may play an important regulatory role in NOD2‐triggered inflammation following cerebral ischaemia injury.

**Figure 7:**
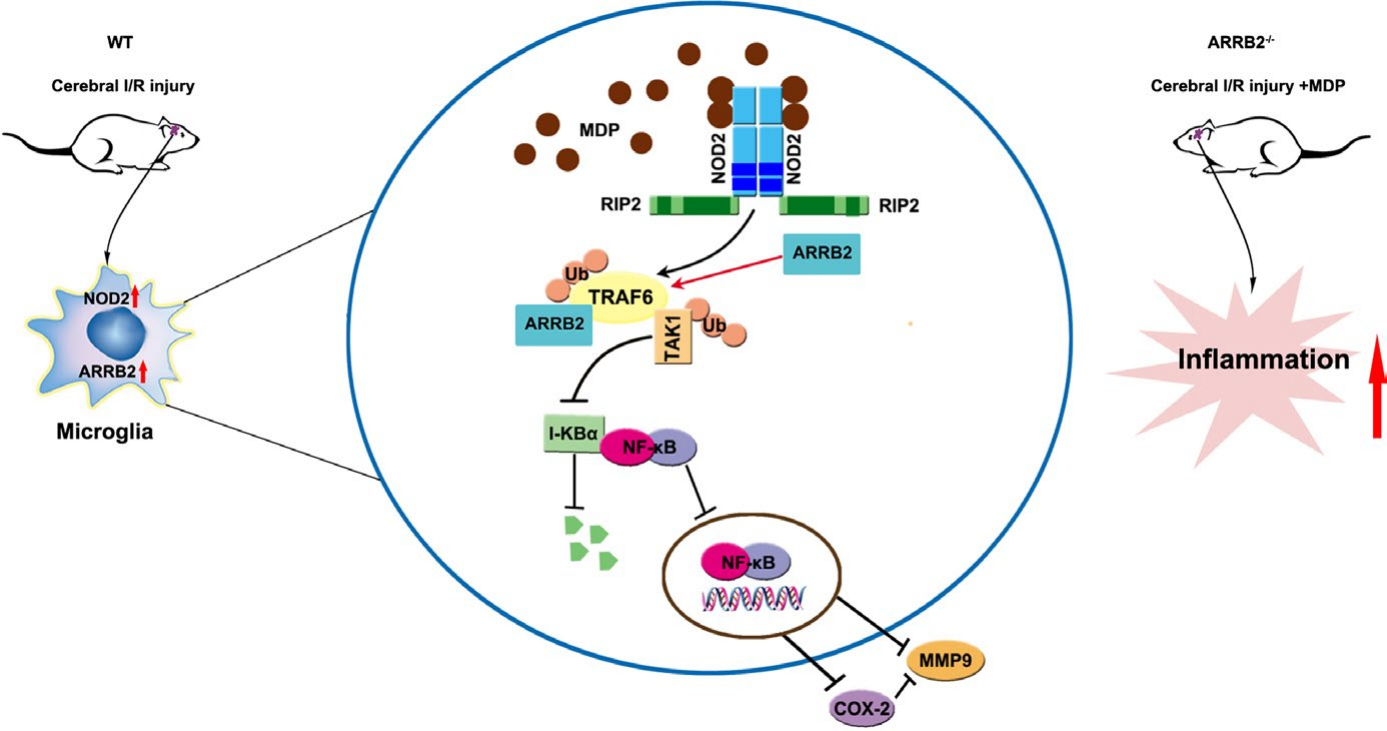
Schematic representation showing that β‐arrestin2 (ARRB2) negatively regulated NOD2 signalling pathway through the association with TRAF6 in cerebral ischaemia/reperfusion injury. MDP stimulation promoted the formation of ARRB2/TRAF6 complex which suppressed NF‐κB/COX‐2/MMP‐9 signalling

In summary, we have found that NOD2 stimulation mediated robust and broad inflammatory response in microglia and ARRB2 negatively regulated NOD2‐induced inflammatory response following ischaemic stroke by combination with TRAF6 (Figure 7). Taken together, ARRB2 emerged as an important control point in the integration of inflammatory responses mediated by NOD2, which may prove useful in the future development of new therapeutic approach for the treatment of ischaemic stroke.

## CONFLICTS OF INTEREST

The authors confirm that there are no conflicts of interest.
